# Autoría de obsequios: un acercamiento a su frecuencia en una revista peruana

**Published:** 2019-09-01

**Authors:** Hugo Arroyo Hernández, Lely Solari

**Affiliations:** Oficina General de Información y Sistemas, Instituto Nacional de Salud, Lima, Perú Oficina General de Información y Sistemas Instituto Nacional de Salud Lima Perú; Centro Nacional de Salud Pública, Instituto Nacional de Salud, Lima, Perú Centro Nacional de Salud Pública Instituto Nacional de Salud Lima Perú

Lima, 5 de agosto, 2019 Señores

Comité Editorial Revista *Biomédica*

E. S. D.

Estimados señores:

Leímos con interés el artículo publicado por Zafra-Tanaka, *et al.,* en el número 2 del volumen 39 del 2019 de la revista *Biomédica,* en el cual se describe una aproximación a la denominada autoría de regalo con base en una revisión de las contribuciones de autoría mencionadas en artículos originales de una revista médica peruana ^1^, y consideramos que es necesario hacer algunas precisiones al respecto.

En primer lugar, resulta metodológicamente deseable medir la autoría mediante una encuesta dirigida a los autores. Varios estudios con esta metodología se han publicado y podrían orientar a los autores para realizar un estudio en el futuro que brinde información más precisa y exhaustiva sobre la realidad de la autoría ^2^.

En segundo lugar, en la discusión del estudio se menciona como parte de sus limitaciones que las contribuciones de autoría en la revista no serían reales debido a que “[…] los editores de la revista pueden haber descartado algunos manuscritos por no cumplir con los criterios de autoría, lo cual nos indicaría que el problema es mucho mayor al hallado en este estudio […]”. Esto resulta una suposición muy lejana de la realidad. Debemos manifestar que los editores no podemos descartar manuscritos por sus reportes de autoría y la publicación de las contribuciones de cada autor; las publicaciones científicas siempre se han regido por la buena fe y, aunque nuestra revista fomenta decididamente el cumplimiento de los criterios de autoría del *International Committee of Medical Journal Editors* (ICMJE), respeta la información original brindada por los autores. Ante una solicitud para excluir o incluir autores, los editores no pueden ser árbitros o determinar qué proceso cumplió o no cada uno de los autores ^3^. Por lo tanto, se siguen flujogramas de decisión establecidos por el *Committee on Publication Ethics* (COPE), según los cuales el proceso editorial del manuscrito se detiene hasta que los involucrados expresen conformidad con los cambios realizados en la autoría y, de no llegar a un acuerdo unánime, la institución o las instituciones donde se llevó a cabo la investigación deben ser quienes lo determinen ^4^.

En tercer lugar, nos parece importante poner los hallazgos en el contexto adecuado. En la publicación de Wislar, citada por los autores, se aprecia como un gran logro el haber disminuido a 21 % la autoría honoraria en varias revistas con alto factor de impacto, muchas de ellas consideradas como ‘revistas *top*’. Los problemas de autoría no son, entonces, propios de nuestra revista o nuestro país y existen en las mejores publicaciones internacionales ^2^. Frente a estos problemas, en la actualidad hay nuevas propuestas de autoría como la contenida en *The Structured Contributor Role Taxonomy* (CRediT), que, además de dar transparencia y reconocimiento de las contribuciones de los investigadores, aspira a dar información más precisa sobre las contribuciones de cada investigador y permite identificar revisores y especialistas que han tenido una variedad de roles en la investigación, lo cual permite una visión más holística de sus contribuciones a los resultados de los estudios ^5^^,^^6^.

Los editores de revistas se enfrentan con frecuencia a problemas de autoría que se manejan antes de la publicación de un artículo y, en general, las buenas prácticas en las publicaciones científicas de ciencias de la salud deben ser impulsadas de todas las maneras posibles. Sin embargo, parte del proceso de mejoramiento debería incluir una práctica metodológicamente exhaustiva en las publicaciones relacionadas con bibliometría, para evitar puntos de vista que pudieran crear visiones negativamente sesgadas sobre nuestras revistas.

## References

[1] Zafra-Tanaka JH, Roca C, Canari-Casano JL, Vargas-Calla A (2019). Autoría de regalo: una aproximación a su frecuencia en una revista peruana. Biomédica.

[2] Wislar JS, Flanagin A, Fontanarosa PB, Deangelis CD (2011). Honorary and ghost authorship in high impact biomedical journals: A cross sectional survey. BMJ.

[3] International Committe of Medical Journal Editors, ICMJE (2018). Defining the role of authors and contributors.

[4] Committee on Publication Ethics, COPE How to recognize potential authorship problems.

[5] Holcombe A (2019). Farewell authors, hello contributors. Nature.

[6] Allen L, O’Connell A, Kiermer V (2019). How can we ensure visibility and diversity in research contributions? How the Contributor Role Taxonomy (CRediT) is helping the shift from authorship to contributorship. Learned Publishing.

