# Supplementary material for: Gift authorship: An approach to its frequency in a Peruvian journal
Source: Biomedica. 2019 Sep 1;39(3):611–2. [Article in Spanish] (PMC7357357)
Supplement: Related texts

# Reply: Respuesta a carta del editor

**Authors:** Jessica Zafra Tanaka

Lima, 21 de agosto, 2019

Señores Comité Editorial

Revista *Biomédica*

Estimados señores:

Hemos leído la carta escrita por los editores de la *Revista Peruana de Medicina Experimental y Salud Pública* y consideramos que no tenemos más que agregar. Por ese motivo vamos a optar por no responderla.
